# TLR1/2 and 5 induce elevated cytokine levels from rheumatoid arthritis monocytes independent of ACPA or RF autoantibody status

**DOI:** 10.1093/rheumatology/keaa220

**Published:** 2020-06-28

**Authors:** Ryan S Thwaites, Sarah Unterberger, Giselle Chamberlain, Karen Walker-Bone, Kevin A Davies, Sandra Sacre

**Affiliations:** Brighton and Sussex Medical School, University of Sussex, Brighton, UK

**Keywords:** rheumatoid arthritis, toll-like receptor, interleukin-6, RF, ACPA, TNFα, interleukin-10, DAS28

## Abstract

**Objective:**

RA is an autoimmune inflammatory joint disease. Both RF and ACPA are associated with more progressive disease and higher levels of systemic inflammation. Monocyte activation of toll-like receptors (TLRs) by endogenous ligands is a potential source of increased production of systemic cytokines. RA monocytes have elevated TLRs, some of which are associated with the disease activity score using 28 joints (DAS28). The aim of this study was to measure TLR-induced cytokine production from monocytes, stratified by autoantibody status, to assess if their capacity to induce cytokines is related to autoantibody status or DAS28.

**Methods:**

Peripheral blood monocytes isolated from RA patients and healthy controls were stimulated with TLR1/2, TLR2/6, TLR4, TLR5, TLR7, TLR8 and TLR9 ligands for 18 h before measuring IL-6, TNFα and IL-10. Serum was used to confirm the autoantibody status. Cytokine levels were compared with RF, ACPA and DAS28.

**Results:**

RA monocytes demonstrated significantly increased IL-6 and TNFα upon TLR1/2 stimulation and IL-6 and IL-10 upon TLR5 activation. TLR7 and TLR9 activation did not induce cytokines and no significant differences were observed between RA and healthy control monocytes upon TLR2/6, TLR4 or TLR8 activation. When stratified by ACPA or RF status there were no correlations between autoantibody status and elevated cytokine levels. However, TLR1/2-induced IL-6 did correlate with DAS28.

**Conclusions:**

Elevated TLR-induced cytokines in RA monocytes were not related to ACPA or RF status. However, TLR1/2-induced IL-6 was associated with disease activity.

**Rheumatology key messages**

- TLR1/2 and TLR5 have an increased capability to induce cytokines in RA monocytes.
- Elevated cytokine production upon TLR activation is not associated with RF or ACPA.
- TLR1/2-induced IL-6 production from monocytes correlates with RA disease activity.

## Introduction

RA is a chronic autoimmune disease, characterized by inflammation of the synovial joints and the production of autoantibodies. Peripheral blood mononuclear cells (PBMCs) infiltrate the synovial membrane releasing cytokines and growth factors that induce proliferation of synoviocytes and the release of matrix metalloproteases leading to the erosion of cartilage and bone [1]. The autoantibodies RF and ACPA are used in the clinical diagnosis of RA. RF is not specific for RA, whereas ACPA are highly specific, often detectable many years prior to disease onset. However, both ACPA and RF are associated with a more severe clinical prognosis alongside increased systemic inflammation, especially when both are present [2, 3]. In recent years, the potential for a pathological role of ACPA has emerged. ACPA have been shown to activate osteoclastogenesis, which may in part explain their association with a more erosive disease progression [4]. Furthermore, ACPA immune complexes containing citrullinated fibrinogen have been shown to activate toll-like receptor (TLR) 4 to induce cytokine production [5].

The potential for TLRs to promote inflammation in RA through activation by endogenous ligands has been recognized for many years. TLRs are a family of innate immune receptors that upon activation induce high levels of pro-inflammatory cytokines. In humans, there are 10 TLRs, most of which have been associated with RA pathogenesis in either experimental or human disease models [6]. Although the majority of studies have focused on TLRs expressed by cells within the inflamed joints, TLR activation of circulating monocytes may also have a role in joint inflammation through migration and differentiation to macrophages and osteoclasts and they may facilitate the systemic inflammation associated with RA co-morbidities such as cardiovascular disease. Indeed, some endogenous TLR ligands are elevated in RA patient serum, such as high mobility group box-1 (HMGB1), which can activate TLR2, TLR4 and TLR5 [7–9]. Furthermore, TLR2, TLR7, TLR8 and TLR9 are expressed at higher levels in RA peripheral blood monocytes, with TLR2 and TLR4 stimulation driving increased cytokine production by monocytes and PBMCs, respectively, from RA patients [10–13]. TLR5 has been shown to induce monocyte chemotaxis and promote differentiation of osteoclast precursor cells into mature osteoclasts [14]. Similarly, activation of TLR7 can lead to differentiation of CD14^+^ monocytes into osteoclasts [15]. In addition, the expression of TLR5 and TLR7 mRNA in RA patient monocytes correlates with the disease activity score using 28 joints (DAS28) [13, 16].

Although both TLR expression in RA monocyte and autoantibody status have been linked with increased inflammation and disease activity, it is not known whether this elevation in the capacity of TLRs to induce cytokines is related to autoantibody status or disease severity. The aim of this study was to measure TLR-induced cytokine secretion by peripheral blood CD14^+^ monocytes and then associate these responses with autoantibody status and DAS28.

## Methods

### Patient samples

The study was approved by the Brighton East Research Ethics Committee (10/H1107/8) and the NRES Committee North West–Lancaster (14/NW/1114). It complies with the Declaration of Helsinki. Patients were diagnosed by a consultant rheumatologist guided by the European League Against Rheumatism/American College of Rheumatology 2010 criteria for RA and recruited during a routine rheumatology clinic assessment. RA patients and healthy controls (HC) were recruited through the Brighton and Sussex University Hospitals Trust. The RA patients were aged on average 56 (range 27–82) years, 69% were female, average mean (s.d.) disease duration was 11.5 (12.3) years, 58% were ACPA positive and 66% were RF positive. DAS28 (CRP) was recorded for 22 of the RA patients, and the scores ranged from 1.17 to 8.68 with a mean (S.d.) of 3.888 (1.878) and a median of 3.633. Of these patients, four were in remission (DAS28 <2.6), four had a low disease activity (DAS28 <3.2), 10 had moderate activity (DAS28 ≤5.1) and four had high activity (DAS28 >5.1). Patients were receiving no DMARDs, steroids or biologic therapies (*n* = 8), taking DMARDs alone (methotrexate or sulfasalazine) (*n* = 7), DMARDs with hydroxychloroquine (*n* = 4), DMARDs with prednisone (*n* = 4), tocilizumab + steroid (*n* = 1), anti-TNFα alone (*n* = 2), anti-TNFα with DMARDs (*n* = 2), anti-TNFα with hydroxychloroquine (*n* = 1) or anti-TNFα with hydroxychloroquine and steroids (*n* = 1). HCs (*n* = 18) were on average 45 (range 26–72) years of age and 83% were female. All participants provided written informed consent.

### Cell culture

Whole venous blood was collected into tubes containing 1.8 mg/ml K_2_EDTA (Becton Dickinson, Oxford, UK). PBMCs were isolated using Ficoll-plaque gradients (Cedarlane, Burlington, ON, Canada) as previously described [17]. Monocytes were isolated from PBMCs using CD14^+^ selection beads (Miltenyi Biotec, Bisley, UK) as per the manufacturer’s instructions before being cultured in RPMI1640 medium supplemented with 5% (v/v) fetal calf serum (PAN-Biotec, Aidenbach, Germany) and 1% (v/v) penicillin–streptomycin solution (PAA, Pasching, Austria). Cells were incubated with or without 100 ng/ml PAM_3_CSK_4_ (PAM3) or 10 ng/ml flagellin (Axxora, Exeter, UK), 1 ng/ml FSL-1, 10 ng/ml lipopolysaccharide (LPS), 2 μg/ml resiquimod (R-848), 2 μM ODN2006 or 2 μM ODN2216 (Invivogen, Toulouse, France) for 18 h at 37°C, 5% CO_2_. The concentrations of the TLR ligands were previously determined by titrating each ligand to determine the threshold for maximal activation.

### Enzyme linked immunosorbent assays

The concentration of IL-6, TNFα and IL-10 were determined by enzyme linked immunosorbent assays (ELISA) (R&D Systems, Abingdon, UK) following the manufacturer’s instructions. Detection was performed using streptavidin–horseradish peroxidase (R&D systems) and a chromogenic 3,3′,5,5′-tetramethylbenzidine substrate (Serascare Life Sciences, Gaithersburg, MD, USA). Absorbance was read and analysed at 450 nm on a spectrophotometric plate reader (BioTek Synergy HT; BioTek, Swindon, UK) using Gen5 version 1.08.4 software (BioTek).

ACPA levels were measured using a cyclic citrullinated peptide-3 IgG ELISA kit (Inova Diagnostics, San Diego, CA, USA) as per the manufacturer’s instructions. RF autoantibody status was determined using a RF IgG ELISA kit (Abnova, Taipei, Taiwan), according to the manufacturer’s instructions.

### Statistics

Mean, S.d., standard error of the mean (S.e.m.) and statistical significance were calculated using GraphPad Prism version 8.4.2 (GraphPad Software Inc., La Jolla, CA, USA). For statistical analysis, parametric data were compared using Student’s two tailed unpaired *t*-test with Welch’s correction. Non-parametric data were compared with a two tailed Mann–Whitney *U*-test. Correlation analysis was performed to compare DAS28 with TLR-induced cytokine levels using a Pearson two-tailed test. S.e.m. was used for pooled experimental data. Significance is shown as ***P *<* 0.001, **P *<* 0.01 and *P *<* 0.05.

## Results

### TLR1/2 and TLR5 activation induced higher cytokine levels in RA monocytes

**Fig. 1.**
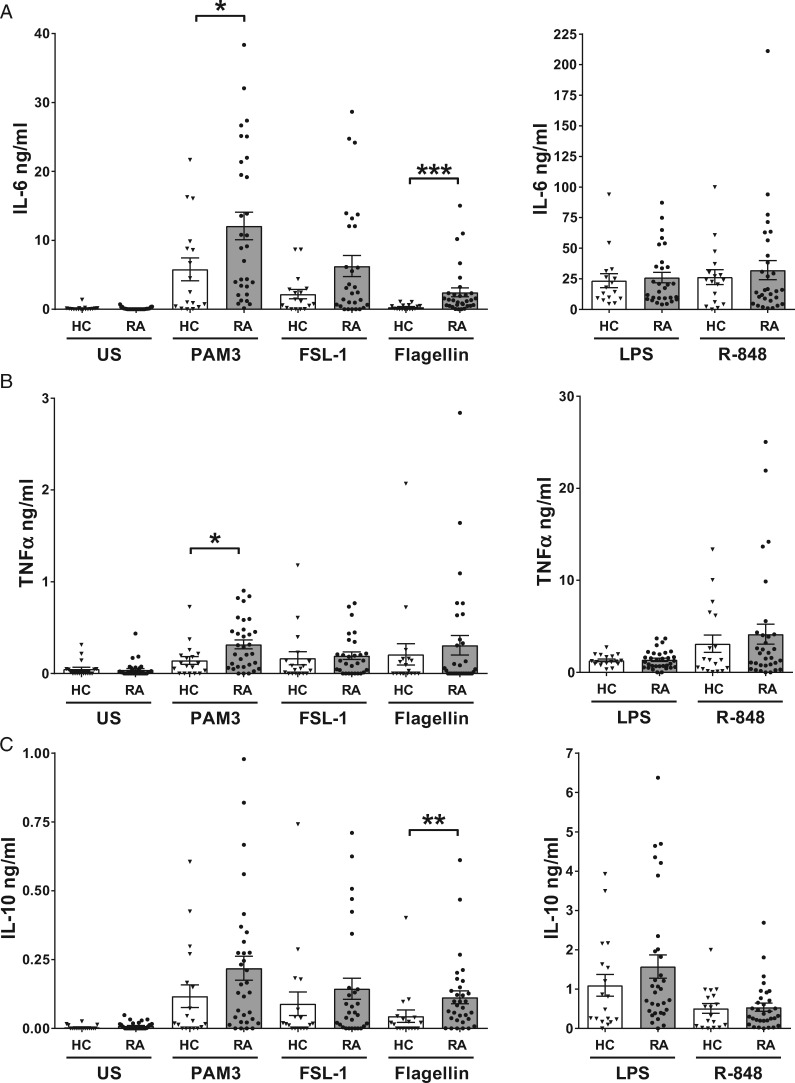
TLR1/2 and TLR5 induce elevated cytokine production by RA monocytes Monocytes from healthy controls (HC) (*n* = 16–18) and RA patients (*n* = 29–32) were either unstimulated (US) or stimulated with 100 ng/ml PAM_3_CSK_4_ (PAM3), 1 ng/ml FSL-1, 10 ng/ml lipopolysaccharide (LPS), 10 ng/ml flagellin or 2 μg/ml resiquimod (R-848) for 18 h. The levels of IL-6 (**A**), TNFα (**B**) and IL-10 (**C**) were measured in the supernatant. ***P < 0.001, **P < 0.01 and *P < 0.05.

TLR2, TLR7, TLR8 and TLR9 are reported to be expressed at higher levels in RA monocytes and both TLR2 and TLR4 can induce elevated cytokine expression in either RA monocytes or PBMCs [10–13]. This suggested the possibility that many of these TLRs could have increased activity in RA monocytes. To determine their activity, the level of cytokine production in RA CD14^+^ monocytes stimulated with TLR1/2, 2/6, 4, 5, 7, 7/8 and 9 ligands was compared with that of HCs. TLR1/2 and TLR5 were found to have elevated responses in RA monocytes (Fig. 1). TLR1/2 activation with PAM3 induced a significantly higher level of IL-6 (*P* *=* 0.0195) and TNFα (*P* *=* 0.0196) with a trend towards higher IL-10 (*P* *=* 0.0639), whereas TLR2/6 with FSL-1 exhibited only a trend towards elevated IL-6 (*P* *=* 0.1536). Stimulation of TLR5 with flagellin produced a significantly higher level of IL-6 (*P* *=* 0.0001) and IL-10 (*P* *=* 0.0016) in RA monocytes but there was no increase in TNFα compared with HC. Activation of TLR2/6 with FSL-1, TLR4 with LPS and TLR7/8 with R-848 induced IL-6, TNFα and IL-10 secretion at a comparable level to HC monocytes (Fig. 1). Stimulation of TLR7 with CL264 or TLR9 with ODN2006 or ODN2216 did not induce any IL-6, TNFα or IL-10. Thus, the response to R-848 (a TLR7/8 ligand) is most probably mediated by TLR8 in human monocytes, as we have previously shown in another study [18].

### Autoantibody status is not associated with higher cytokine production

**Fig. 2:**
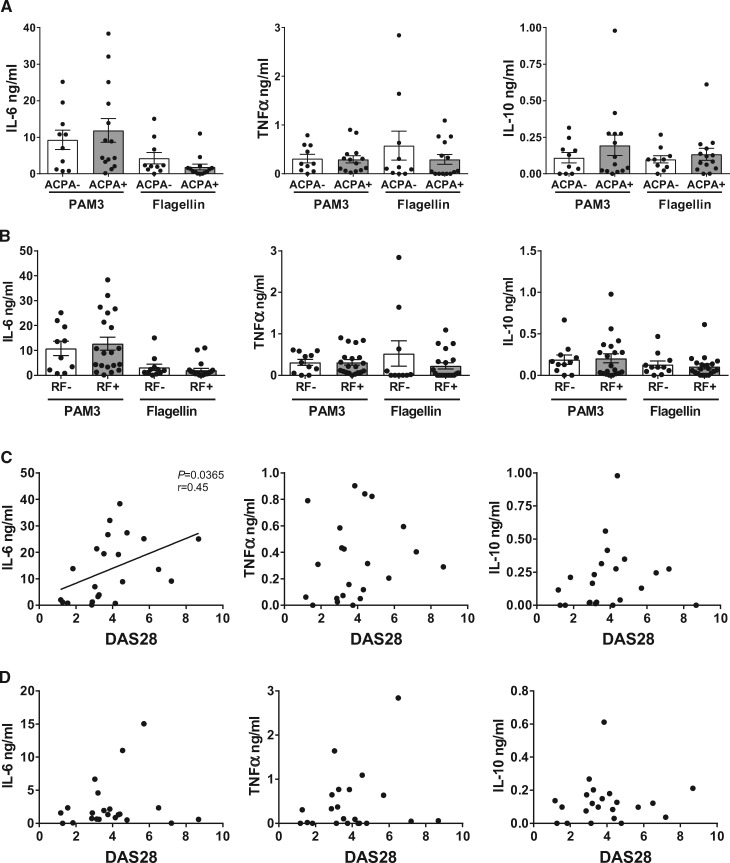
TLR1/2- and TLR5-induced cytokines do not associate with autoantibody status but TLR1/2-induced IL-6 correlates with DAS28. RA monocytes were either unstimulated (US) or stimulated with 100 ng/ml PAM_3_CSK_4_ (PAM3) or 10 ng/ml flagellin for 18 h. IL-6, TNFα and IL-10 were measured in the supernatants and compared by ACPA status (**A**; ACPA+: *n* = 14, ACPA–: *n* = 10) and RF status (**B**; RF+: *n* = 20, RF–: *n* = 10–11). The data were then analysed for association with the disease activity score using 28 joints (DAS28) in PAM3- (**C**; *n* = 22) and flagellin- (**D**; *n* = 22) stimulated monocytes.

Although TLR1/2 and TLR5 could induce higher levels of cytokines in RA monocytes, there was a spread of cytokine levels within the patient samples, with some producing levels comparable to HCs. As both RF and ACPA have been associated with higher levels of systemic cytokines in RA patients, the results were further analysed to explore if there was a relationship between autoantibody status and higher TLR-induced cytokine production [3]. When subdivided by ACPA status, there was no significant difference in the production of IL-6, TNFα or IL-10 upon stimulation of TLR1/2 or TLR5 (Fig. 2A). Similarly, when divided by RF status, there was also no change in the levels of IL-6, TNFα or IL-10 in RA monocytes activated with TLR1/2 or TLR5 ligands (Fig. 2B).

### TLR1/2-induced IL-6 correlates with disease activity

TLR5 mRNA expression had previously been associated with the DAS28 [16]. Thus, to confirm if the increased capacity of TLR1/2 and TLR5 to induce cytokines was correlated with disease activity, the cytokine levels were compared with DAS28. TLR1/2-induced IL-6 was significantly correlated with DAS28 (*P =* 0.0365) but TNFα and IL-10 were not (Fig. 2C). None of the cytokines measured after TLR5 activation significantly correlated with DAS28 (Fig. 2D).

## Discussion

As both autoantibody status and TLRs have been associated with elevated inflammation and disease severity in RA, this study investigated whether there was a link between elevated levels of TLR-induced cytokines in peripheral blood CD14^+^ monocytes and RF or ACPA status. An increased capacity for monocytes to produce pro-inflammatory cytokines in the peripheral blood may reflect early changes in their phenotype prior to infiltrating the synovium and have implications for the development of extra-articular symptoms.

Upon TLR1/2 stimulation, significantly higher levels of IL-6 and TNFα were observed from the RA monocytes. This was not unexpected, as TLR2 has previously been reported to be expressed at a higher level on RA monocytes and stimulation with lipoteichoic acid (a TLR2 ligand that can activate both TLR1/2 and TLR2/6 heterodimers) could induce elevated levels of intracellular cytokines, as shown by flow cytometry [12]. Here, the data confirm this finding and further demonstrate that this elevated response when compared with HC monocytes may be predominantly from the TLR1/2 heterodimer, as TLR2/6 activation (with FSL-1) only displayed a trend towards elevated IL-6.

Interestingly, despite reports of higher expression of TLR4, TLR7, TLR8 and TLR9 in RA monocytes, no difference in cytokine expression was observed between RA and HC monocytes upon activation of these TLRs. In fact, TLR7 and TLR9 did not produce any IL-6, TNFα or IL-10. However, this does not exclude a contribution of these TLRs on monocytes to RA pathogenesis, as they may induce other inflammatory mediators not measured in this study or mediate other effects such as differentiation of CD14^+^ monocytes into osteoclasts, as has been reported for TLR7 [15]. However, our finding that the TLR4 responses were comparable to HC monocytes differs from another study, which demonstrated elevated TLR4-induced IL-6 and TNFα production in leukocytes from early onset RA patients [11]. This difference may partly be due to the larger sample size used in our study and the use of purified CD14^+^ cells rather than a mixed cell population or could reflect differences between early RA and more established disease.

Previously, a greater percentage of CD14^+^ monocytes have been shown to express TLR5 in RA monocytes compared with HC [16]. Although our study demonstrated a significant elevation of IL-6 and IL-10 upon activation of TLR5 in RA monocytes, which may be expected if a greater number of cells are expressing TLR5, the level of TNFα was similar to that of HC monocytes. This suggests that the explanation for the elevated IL-6 and IL-10 may be more complex than a simple increase in receptor expression; instead, there may be more subtle changes in the downstream regulation of TLR5 signalling in RA monocytes. A similar situation has previously been reported in RA synovial macrophages where TLR2 and TLR4 expression did not correlate with increased levels of cytokine production, whereas a correlation was observed in HC macrophages [19]. The explanation for the increased cytokine production from TLR1/2, TLR2/6 and TLR5, but not from TLR4 or TLR8, is unclear. All of these TLRs activate the downstream myeloid differentiation primary response 88 (MyD88) signalling pathway to induce TNF, IL-10 and IL-6, a pathway that is shared by all of the TLR family apart from TLR3 [20]. This would suggest that there could be subtle differences in the regulation of this pathway between individual TLRs that have yet to be identified.

TLR5 mRNA expression has also been shown to correlate with DAS28 in RA monocytes [16]. However, correlation of cytokine production upon TLR1/2 and TLR5 stimulation with DAS28 only highlighted an association between TLR1/2-induced IL-6 and disease activity. Thus, the mechanism related to the increase in TLR5 expression may be independent of the mechanism underlying the amplification in cytokine production. A limitation of this study is that the data were generated using monocytes from patients receiving a mixed range of treatment combinations, with only eight patients not receiving any DMARDs, steroids or biologic therapies. The DAS28 was not recorded for all of the patients in this study and thus it was not possible to correlate DAS28 with cytokine production in the patients not receiving these therapies. It is also possible, that some of the therapies could have affected the level of TLR-induced cytokine production. However, a preliminary analysis of the data stratified by those receiving treatment compared with those who were not, produced similar results in both groups suggesting that the underlying changes in downstream TLR signalling in the RA patients are not affected by therapy (data not shown).

Overall the data presented in this study demonstrate that TLR1/2- and TLR5-induced cytokine secretion is elevated in RA monocytes but this increased capacity for cytokine production is independent of autoantibody status with only IL-6 induced by TLR1/2 associated with disease activity.
